# Antagonism of cadmium-induced liver injury in ducks by α-bisabolol

**DOI:** 10.3389/fvets.2022.1024549

**Published:** 2022-11-07

**Authors:** Sara T. Elazab, Walter H. Hsu

**Affiliations:** ^1^Department of Pharmacology, Faculty of Veterinary Medicine, Mansoura University, Mansoura, Egypt; ^2^Department of Biomedical Sciences, College of Veterinary Medicine, Iowa State University, Ames, IA, United States

**Keywords:** α-bisabolol, cadmium, hepatotoxicity, inflammation, oxidative stress

## Abstract

Cadmium (Cd) is an ecological pollutant which causes hazardous effects in animals and humans. The aim of this study was to investigate the role of α-bisabolol (BISA) in antagonizing the Cd-induced hepatotoxicity in ducks. Two-week old ducks were allocated into 8 groups (10 ducks/group): Group I received basal diet and was gavaged with sunflower oil (BISA vehicle, 1.1 mL/kg/day); group II was administered BISA orally (50 mg/kg/day; diluted with sunflower oil); groups III, IV, and V were fed the basal diet mixed with CdCl_2_ at 37.5, 75, and 150 mg/kg diet, respectively, and were gavaged with sunflower oil; group VI, VII, and VIII were given basal diet containing CdCl_2_ at the aforementioned consecutive doses plus BISA. All treatments were provided daily for 4 weeks. Exposure to CdCl_2_ induced mortality in ducks, increased hepatic Cd content and serum levels of hepatopathic biomarkers, and caused oxidative stress and morphological alterations in ducks' liver. Furthermore, exposure to Cd caused upregulation of the mRNA of proinflammatory cytokine tumor necrosis factor-α and apoptotic gene Bax, and that of cyclooxygenase-2 protein in the liver. All effects of Cd were dose-dependent. BISA antagonized all of the aforementioned CdCl_2_-induced changes. These findings suggested that BISA exert the hepatoprotective effect against Cd toxicity through reducing the hepatic content of Cd as well as antagonizing oxidative insults, inflammation, and apoptosis. Thus, BISA has a great potential to be used as an antidote in the control of Cd poisoning.

## Introduction

Cadmium (Cd), a heavy metal, is considered one of the deleterious environmental pollutants. Cd is one of the 10 most hazardous compounds to human and animal health (1). In 1993, the International Agency for Research on Cancer ranked Cd as a Class I carcinogen (2, 3). The sources of Cd exposure stem from its industrial utilization as plastic stabilizers, pigments, alloy, and batteries, as well as being used in mining process (4). Additionally, the soil pollution by Cd results from the extensive usage of fertilizers, pesticides, waste water, and sewage sludge (5). Cd is highly water soluble which leads to its rapid absorption by plants with consequent accumulation in food and feed (6). Former reports have revealed that the bioaccumulation of Cd in chickens can result in serious injuries of several organs, e.g., liver, kidney, heart, lung, brain, testis, and ovary (7). Since Cd has a long half-life of 10–30 years and a slow excretion from the body, it is accumulated in multiple tissues and organs, hence threatening human health *via* transmission through food chain (8).

Oxidative stress is regarded as the chief mechanism for Cd hepatotoxicity (9). Cd enhances the generation of reactive oxygen species (ROS) as hydroxyl radical (OH^−^), hydrogen peroxide (H_2_O_2_), and superoxide (${\text{O}}_{2}^{-}$) which interact with multiple cellular molecules, resulting in lipid and protein peroxidation and DNA injury as well as apoptosis (10).

Numerous investigations have indicated that antioxidant substances exert their ameliorative effects against Cd-induced hepatic damage through their potential role in free radicals scavenging (11). α-Bisabolol (BISA), also named levomenol, is a volatile sesquiterpene monocyclic alcohol (12). It is a natural component of the essential oil of multiple medicinal plants of the *Asteraceae* family (13). BISA has many biological effects, e.g., antimicrobial (14), antioxidant (15), anti-inflammatory (16), anticancer (17), and antimutagenic activities (18). Furthermore, prior investigations highlighted the palliative role of BISA against isoproterenol-induced myocardial infarction in rats (19), neurotoxicity induced by rotenone in *Drosophila melanogaster* (20), and ischemic reperfusion–induced acute renal injury (21). To the best of our knowledge, the effect of BISA on Cd-induced hepatic damage has not been reported.

The present study was performed to assess the antagonistic effect of BISA on Cd-induced hepatotoxicity. In the present research, we used ducks as an experimental model as they are considered as an important experimental animal in environmental toxicology (22). Moreover, ducks are important in animal production in view of their extensive utilization as traditionally available economic animals. Therefore, this study may confer a practical approach to counteract the global Cd pollution.

## Materials and methods

### Chemicals

Cadmium chloride (CdCl_2_) and α-bisabolol were procured from Sigma Aldrich Co. (St. Louis, MO. USA). BISA was used after being diluted with sunflower oil (50 mg BISA/mL sunflower oil). Perchloric acid and nitric acid were bought from Merck Co. (Darmstadt, Germany).

### Experimental animals

In this study, a total of 80 one-day-old Muscovy ducks were provided by Faculty of Agriculture, Mansoura University, Egypt; they were housed in disinfected floor pens covered with sawdust litter. The ambient temperature was set at 30–32°C during the first 7 days and gradually declined to 25 ± 1°C at the end of the study. The environmental humidity was set at 50–65%. The ducks underwent a 2-week adaptation period prior to the study. Throughout the experiment, feed and water were freely available to ducks. The feed ingredients and chemical components of the basal diet are shown in Supplementary Table S1. All procedures conducted in the present work were accepted by the Medical Research Ethics Committee of the Faculty of Veterinary Medicine, Mansoura University, Mansoura, Egypt (Approval No. R/117).

### Experimental design

A total of 80 two-week old ducks were randomly assigned to 8 groups, with 10 ducks in each group. Group I (control group) was offered basal diet and was gavaged with sunflower oil (BISA vehicle) at a dose of 1.1 mL/kg each day (23). Group II was provided with basal diet and received BISA (50 mg/kg/day), in sunflower oil (50 mg BISA/mL), by gavage. The dose of BISA was selected based on previous reports (24, 25). Groups III, IV, and V were fed basal diet containing CdCl_2_ at 37.5, 75, and 150 mg/kg diet, respectively, and were gavaged with sunflower oil at 1.1 mL/kg/day. These CdCl_2_ doses were previously used for toxicological studies in chickens (26, 27). Groups VI, VII, and VIII were fed basal diet plus CdCl_2_ at the above-mentioned consecutive doses and were administered orally BISA (50 mg/kg/day). This experiment lasted 4 weeks, during which the ducks were monitored for clinical signs of Cd-toxicity and weekly body weight changes and mortality. The experimental period was selected according to a former study (28). At the end of the 4th week, blood samples from 6 ducks in each group were collected from the wing vein. The serum was obtained *via* centrifugation of the clotted blood samples at 3,000 × *g* for 10 min, and stored at −80°C until the evaluation of liver function biomarkers. The ducks were then euthanized by exsanguination and the liver was dissected and washed with ice-cold 0.9% NaCl solution. The liver tissue was divided into 3 portions; the first portion was homogenized, then centrifuged at 3,000 × *g* for 15 min and the harvested supernatant was kept at −80°C for later determination of oxidative stress biomarkers. The second portion was kept at −80°C to be used in the quantitative real time polymerase chain reaction (qRT-PCR). The third portion was fixed in 10% formalin for histopathological and immunohistochemical evaluations.

### Biochemical investigation

#### Serum hepatic indices

The activity of serum alanine aminotransferase (ALT) and total bilirubin were assayed spectrophotometrically using commercial kits purchased from Randox Co. (Crumlin, UK) and BioMed Co. (Cairo, Egypt), respectively. The procedures were performed by following the instructions provided by manufacturers.

#### Antioxidant and oxidative stress parameters

The malondialdehyde (MDA) level in the hepatic tissue homogenate was evaluated as previously described by Ohkawa et al. (29). The antioxidant enzymatic activities of catalase (CAT) and superoxide dismutase (SOD) were determined using a spectrophotometer following the reports of Aebi (30) and Minami and Yoshikawa (31), respectively. In addition, the content of reduced glutathione (GSH), the nonenzymatic antioxidant marker, was evaluated as described by Paglia and Valentine (32).

### Determination of hepatic Cd content

One gram of liver sample was excised and subjected to digestion by a mixture of 2.5 mL perchloric acid (70%) and 5 mL nitric acid (65%). After incubation of this mixture overnight in a water bath at 53°C, the suspension was filtered and 30 mL deionized water was added to the filtrate for dilution. The hepatic Cd concentrations were determined using flame atomic absorption spectrophotometer at a wavelength of 228.8 nm (Buck Scientific 210 VGP, Inc.) following the technique recommended by the Association of Official Analytical Chemists (33).

### Transcription of target genes [tumor necrosis factor-α (TNF-α), Bax, and Bcl-2] utilizing qRT-PCR)

The hepatic RNA was isolated utilizing QIAamp RNeasy Mini kit (Qiagen, Heidelberg, Germany). A NanoDrop^®^ (ND-1000) was used for measuring the purity and concentration of the extracted RNA. The first strand of cDNA was synthesized from the isolated RNA employing QuantiTect Reverse Transcription kit (Qiagen, Heidelberg, Germany) according to manufacturer's guidelines. qRT-PCR reactions were performed using SYBR Green QuantiTect PCR kits (Qiagen, Heidelberg, Germany) by the aid of a Rotor-Gene Q cycler RT-PCR equipment (Qiagen, Heidelberg, Germany). β-Actin was used as the reference housekeeping gene. Primer sequences of the investigated duck genes were derived from a previous study (32) and are listed in Table 1. The cycling conditions of qRT-PCR were as follows: 94°C for 5 min; 30 cycles of 94°C for 30 s, 55°C for 30 s and 72°C for 60 s, at last elongation at 72°C for 10 min. The relative expression levels of the target genes were determined *via* the comparative 2^Δ*ΔCt*^ method (Ct: cycle threshold) as reported by Yuan et al. (35).

**Table 1:** Sequences of primers utilized in qRT-PCR.

**Target gene**	**Forward primer (5^′^-3^′^)**	**Reverse primer (5^′^-3^′^)**	**References**
TNF-α	CCGTGGTCAGTTT7gCCATCAGG	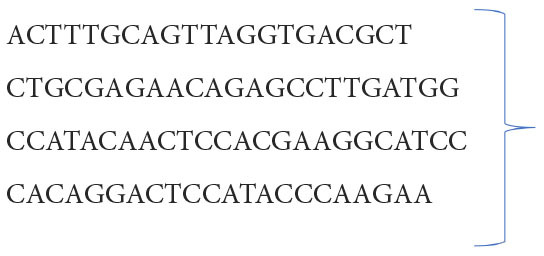	
Bax	GCGGACGGAGCCTTCAACTG		(34)
Bcl-2	GAGTTCGGCGGCGTCATGTG		
ß. actin	ATGTCGCCCTGGATTTCG		

### Hepatic histopathological screening

Tissue specimens from liver were preserved in 10% formalin. Thereafter, they were processed following the classical histological technique, starting with dehydration using ascending concentrations of alcohol and ended by embedding in paraffin. Multiple tissue sections (4 μm in thickness) were prepared for histological staining. The sections were stained with hematoxylin and eosin (H&E) and then investigated under a light microscope as reported by Bancroft and Layton (36). We conducted semiquantitative scoring of hepatic lesions following the method of Gibson-Corley et al. (37). Lesions in 3 fields chosen randomly from each slide for each duck were determined. A blinded process was utilized for lesion scoring [Score scale: 0 = normal with 0% lesions; 1 = ≤ 25%; 2 = 26–50%; 3 = 51–75%; 4 = 76–100%]. The identification of hepatic lesions in ducks depended on the ratio of hepatocyte degeneration. congestion and fibrosis. The extent of fibrosis was examined by staining the liver sections with Masson's trichrome stain. The sections were photographed and investigated using a light microscope. Quantification of the percent of the area with fibrosis was conducted using Image J software (National Institutes of Health, Bethesda, MD, USA).

### Immunohistochemistry

Cyclooxygenase-2 (COX-2) immunohistochemical staining of ducks' hepatic sections was conducted utilizing the avidin-biotin peroxidase method as reported by Lin and Prichardas (38). Photomicrographs of the sections were taken employing a digital camera (Leica EC3, Leica, Leica 148 Germany) attached to a microscope (Leica DM500, Leica, Germany). The Image J software (National Institutes of Health, Bethesda, MD, USA) was used to quantify the intensities of immunostaining. The inverse mean density was evaluated as reported by Vis et al. (39) in 3 fields being selected in a random method from different sections of 6 ducks/group.

### Statistical analysis

All values are expressed as mean ± SEM. The Shapiro Wilk test was applied for examining the normality of the data. Statistical analysis was achieved using Statistical Package for Social Sciences (SPSS), version 20 for windows (SPSS Inc., Chicago, IL, USA). Data of histopathological score and immunohistochemical examination were investigated utilizing Kruskal-Wallis followed by Dunn's test to compare all means. All other data were analyzed employing the one-way ANOVA. Mean comparisons were performed utilizing Tukey's *post-hoc* test. The *p* < 0.05 was regarded as statistically significant.

## Results

### Clinical signs observations, body weight changes and mortality rates

Several clinical signs of Cd toxicity were observed in ducks receiving CdCl_2_ at 75 and 150 mg/kg such as dullness, gasping, restlessness, depression, lethargy, increased water consumption, weakness, and imbalanced gait. These clinical signs began to emerge during the 2nd week of the experiment and aggravated by the end of the 4th week. These signs were more prominent in the group receiving CdCl_2_ at 150 mg/kg feed. On the other hand, ducks in BISA + Cd 75 and BISA + Cd 150 groups displayed milder clinical signs and reduced water consumption. Furthermore, CdCl_2_-treated groups exhibited a significant reduction (*p* < 0.05) in the final body weight (FBW) and body weight gain (BWG), compared to the control group. Meanwhile, a significant elevation (*p* < 0.05) in FBW and BWG was observed in groups receiving BISA + CdCl_2_, compared to those receiving CdCl_2_ only. The mortality rates of CdCl_2_-treated ducks at doses of 75 was 20% as one duck died in the 1st week of experiment and another duck in the 3rd week. Moreover, the mortality rate of ducks receiving CdCl_2_ at 150 mg/kg was 30% as one duck died in the 1st week, another one died in the 2nd week, and the third duck died in the 4th week. While, the control group had 0% mortality. Contrarily, the mortality rates in BISA + Cd 75 and BISA + Cd 150 groups were 0 and 10% (as one duck died from BISA + Cd 150 in the 2nd week), respectively, which were lower than those in the groups receiving CdCl_2_ only at the corresponding dose (Table 2).

**Table 2:** Effect of α-bisabolol on body weight changes and mortality in ducks receiving CdCl_2_ in feed for 4 weeks.

**Experimental groups**	**Parameters**			
	**IW (g /duck)**	**FBW (g/duck)**	**BWG (g/duck)**	**Mortality**
C	51.52 ± 0.26	1814.00 ± 20.99^a, b^	1762.48 ± 19.45^a, b^	0/10
BISA	51.46 ± 0.19	1853.83± 16.23^a^	1802.37 ± 16.11^a^	0/10
Cd 37.5	51.93 ± 0.23	1572.00 ± 22.15^c^	1520.07 ± 22.03^c^	0/10
Cd 75	52.03 ± 0.18	1368.50 ± 34.92^d^	1316.47 ± 34.99^d^	2/10
Cd 150	51.56 ± 0.22	1207.67 ± 36.47^e^	1156.10 ± 36.41^e^	3/10
BISA + Cd 37.5	51.75 ± 0.40	1723.33 ± 27.37^b^	1671.58 ± 27.61^b^	0/10
BISA+ Cd 75	52.08 ± 0.28	1636.50 ± 37.53^c^	1584.42 ± 37.73^c^	0/10
BISA + Cd 150	51.8 ± 0.16	1545.00 ± 27.06^c^	1493.20 ± 27.02^c^	1/10

Data of body weight changes are presented as the mean ± SEM (n = 6).

a−e Different superscripts within each column (for each parameter) represent significant differences (p < 0.05). C, control, BISA; α-bisabolol; Cd 37.5, Cd 75, Cd150: CdCl_2_ was administered at 37.5, 75, and 150 mg/kg feed, respectively, for 4 weeks); IW, initial weight; FBW, final body weight; BWG, body weight gain.

### Cd content in hepatic tissues

**Figure 1:**
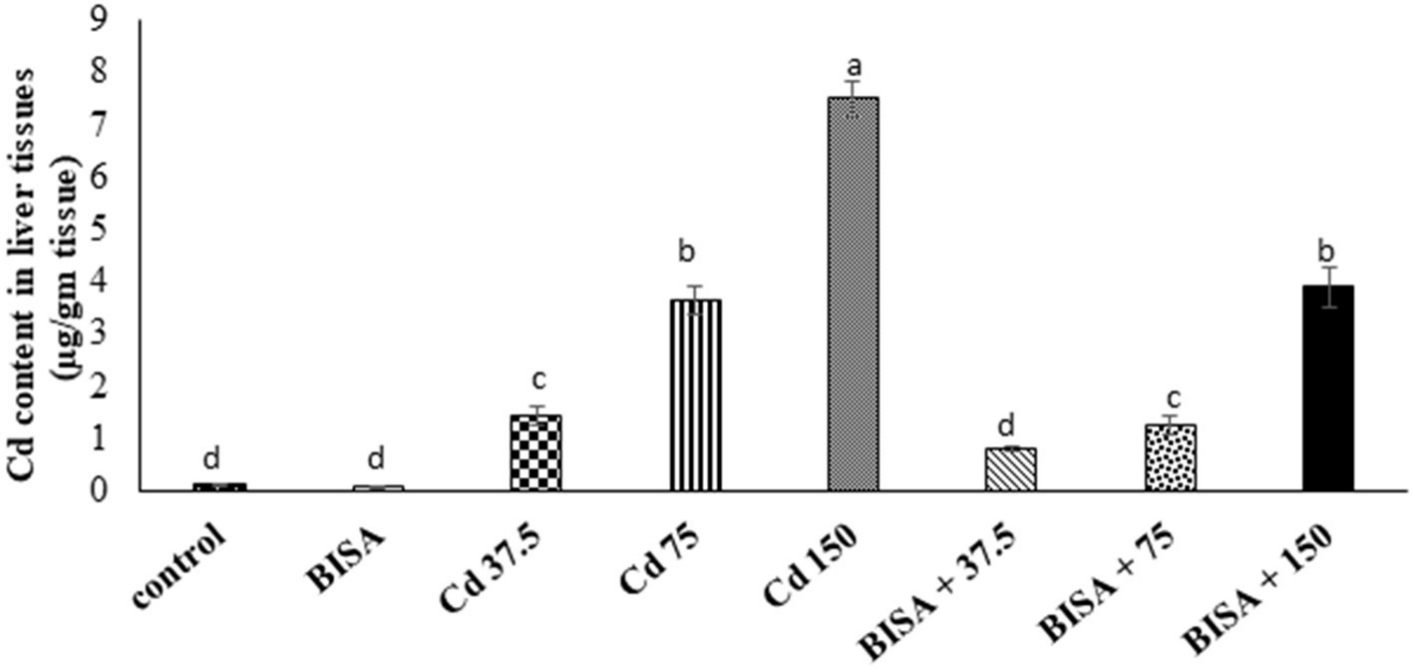
Concentration of Cd in the hepatic tissue of ducks following treatment with oral α-bisabolol (50 mg/kg/day) and/or CdCl_2_ at 37.5, 75, and 150 mg/kg diet, respectively) for 4 weeks. Data are shown as mean ± SEM (*n* = 6 ducks). Bars carrying different letters are significantly different from one another (*p* < 0.05). C, control; BISA, α-bisabolol.

As illustrated in Figure 1, the hepatic Cd content in CdCl_2_-exposed ducks at doses of 37.5, 75, and 150 mg/kg diet were significantly higher (*p* < 0.05) than that in the controls. In contrast, a significant reduction (*p* < 0.05) in the hepatic Cd content was recorded in all BISA + Cd groups, compared to those treated with the corresponding CdCl_2_ dose only.

### Serum hepatic parameters

BISA alone did not cause significant alterations in the hepatic indices ALT and bilirubin. On the other hand, serum ALT activity and total bilirubin level significantly (*p* < 0.05) increased in a dose-dependent manner in CdCl_2_-treated groups (III, IV, and V) by 70, 108, and 193%, respectively (for serum ALT) and by 31, 86, and 186%, respectively (for serum total bilirubin), as compared to the control group. However, co-administration of BISA with CdCl_2_ at different CdCl_2_ doses (groups VI, VII, and VIII) showed a significant (*p* < 0.05) decline in serum ALT activity and total bilirubin level as compared to ducks exposed to CdCl_2_ alone at the corresponding dose. These hepatic indices returned to normal in BISA + Cd 37.5 and BISA + Cd 75 groups, while they were still higher in BISA + Cd 150 group than the control group, but lower than the CdCl_2_ alone group (Table 3).

**Table 3:** Effect of α-bisabolol on serum ALT and bilirubin levels in ducks receiving CdCl_2_ in feed for 4 weeks.

**Experimental groups**	**Parameters**	
	**ALT (U/L)**	**Total bilirubin (mg/dL)**
C	19.85 ± 0.94^d^	0.49 ± 0.02^c^
BISA	18.15 ± 0.98^d^	0.41 ± 0.03^c^
Cd 37.5	33.90 ± 1.93^b, c^	0.64 ± 0.05^b, c^
Cd 75	41.47 ± 3.50^b^	0.90 ± 0.06^b^
Cd 150	58.25 ± 4.24^a^	1.39 ± 0.16^a^
BISA + Cd 37.5	23.65 ± 1.57^d^	0.50 ± 0.03^c^
BISA+ Cd 75	25.05 ± 2.47^c, d^	0.55 ± 0.03^c^
BISA + Cd 150	36.85 ± 3.62^b^	0.86 ± 0.05^b^

Data are expressed as the mean ± SEM (n = 6).

a−d Different superscripts within each column (for each parameter) represent significant differences (p < 0.05). C, control, BISA; α-bisabolol; Cd 37.5, Cd 75, Cd150: CdCl_2_ was administered at 37.5, 75, and 150 mg/kg feed, respectively, for 4 weeks), ALT, alanine aminotransferase.

### Histopathological findings

**Figure 2:**
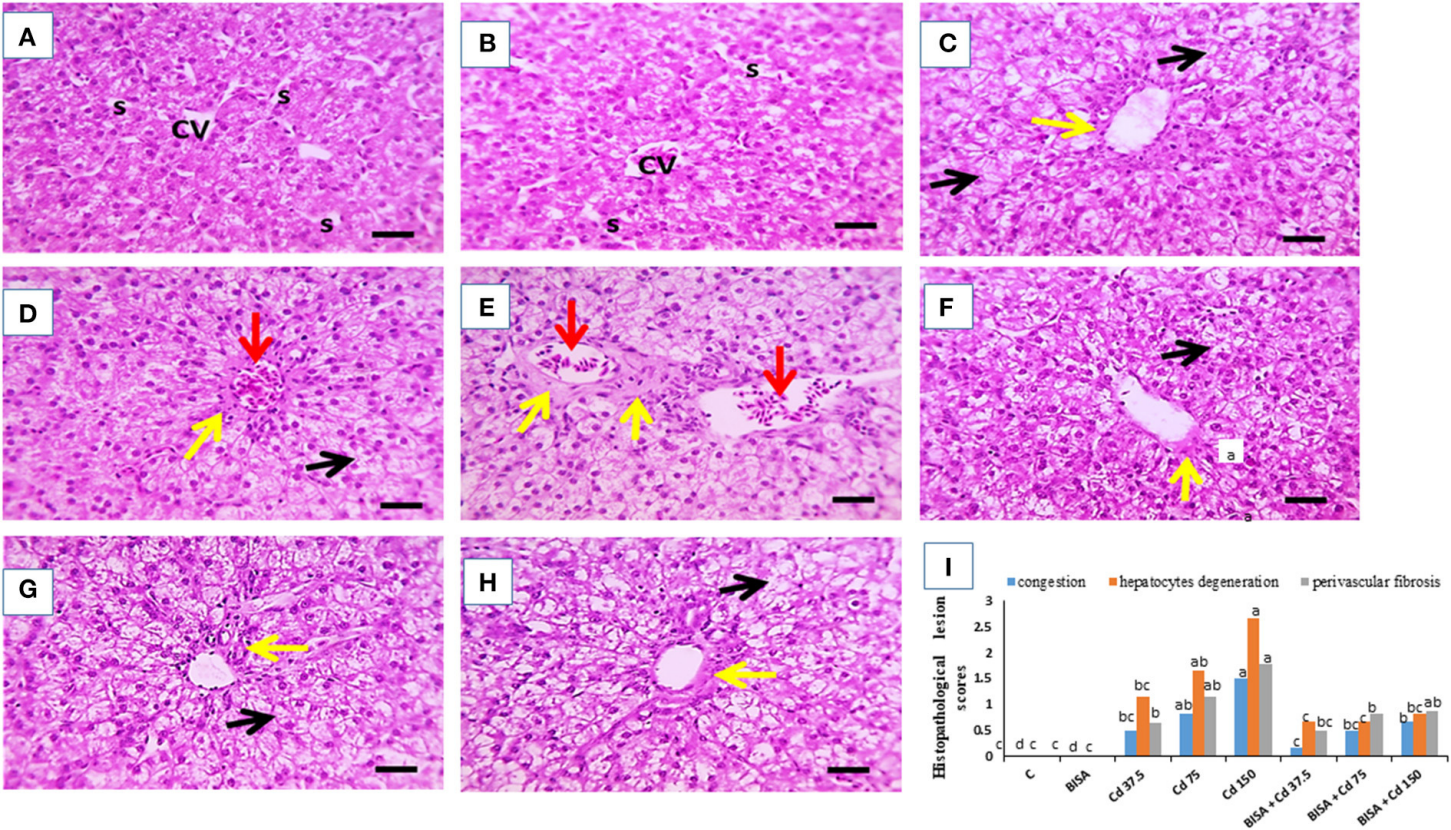
Effect of oral α-bisabolol (BISA; 50 mg/kg/day) on histopathological alterations caused by CdCl_2_ at different doses in ducks' hepatic tissue. **(A)** is for the control group (C), and **(B)** is for the BISA alone group; both groups show normal hepatocytes (H), central vein (CV), and opened sinusoids (S). **(C–E)** represent groups receiving CdCl_2_ at 37.5, 75, and 150 mg/kg diet, respectively, and showing hydropic degeneration in hepatocytes (black arrows), occluded sinusoids, congested central veins (red arrows), perivascular fibrosis (yellow arrows). The higher the dose of CdCl_2_, the more severe the lesions. **(F–H)** are for groups receiving BISA + CdCl_2_ at 37.5, 75, and 150 mg/kg diet, respectively, exhibiting less severe lesions than those of the corresponding Cd alone groups. **(I)** shows the semiquantitative scoring of hepatic lesions. Lesions in 3 fields chosen randomly from each slide for each duck were determined. A blinded process was utilized for lesion scoring [Score scale: 0 = normal with 0% lesions; 1 = ≤ 25%; 2 = 26–50%; 3 = 51–75%; 4 = 76–100%]. The identification of hepatic lesions in ducks depended on the ratio of hepatocyte degeneration. congestion and fibrosis. Bars carrying different letters are significantly different from one another (*p* < 0.05). Scale bar = 50 μm. The sections were stained with H&E, X400.

Figure 2 shows the light photomicrographs of hepatic tissues. Figures 2A,B display normal hepatocytes arranged in cords around the central vein with opened sinusoids in the control and BISA groups. In contrast, a dose-dependent histological deformities in the form of hydropic degeneration in hepatic cells, occluded sinusoids, congested central veins, and perivascular fibrosis were detected in CdCl_2_-exposed groups receiving 37.5, 75, and 150 mg/kg diet; the most extreme lesions were seen in CdCl_2_ 150 mg/kg diet-treated ducks (Figures 2C–E). While, the hepatic sections of groups treated with BISA + CdCl_2_ revealed much less severe lesions than the groups receiving the corresponding CdCl_2_ dose alone (Figures 2F–H). The semiquantitative scoring of hepatic lesions is illustrated in Figure 2I.

**Figure 3:**
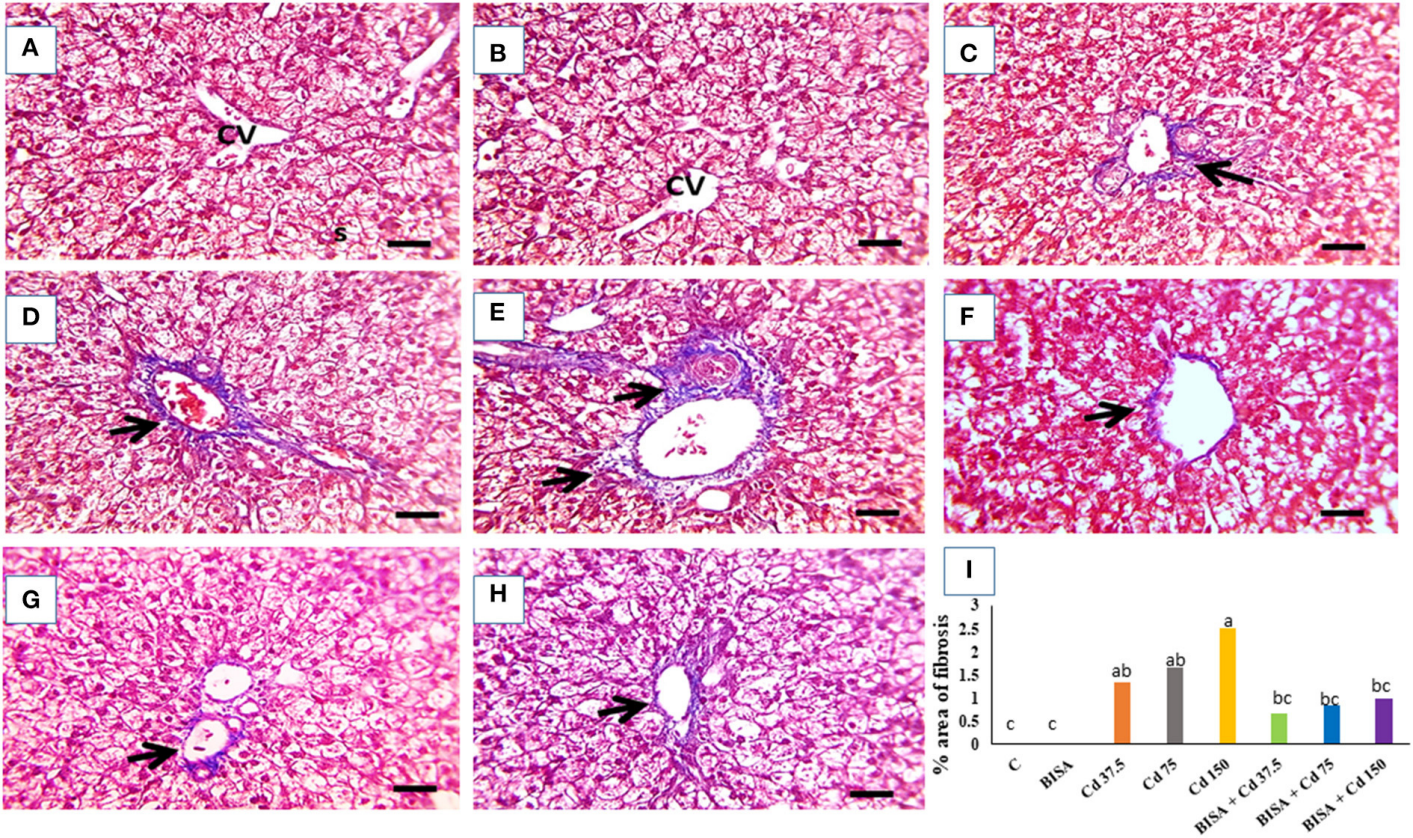
The representative light photomicrographs of ducks' hepatic section (stained with Masson's trichrome, X400). **(A)** Represents the control group (C) and **(B)** is for the α-bisabolol (BISA) alone group; both groups show normal hepatocytes and central vein (CV) with no collagen deposition. **(C–E)** are for groups receiving CdCl_2_ at 37.5, 75, and 150 mg/kg diet, respectively, revealing blue-stained collagen deposition in portal areas which increase with the increasing dose of CdCl_2_. **(F–H)** Represent BISA + Cd 37.5, BISA + Cd 75, and BISA + Cd 150 groups, respectively, and show remarkable reduction in blue color-stained collagen deposition, compared to the corresponding groups receiving CdCl_2_ only. **(I)** Exhibits the percent of area of collagen deposition. Data are shown as mean ± SEM (*n* = 6 ducks). Bars carrying different letters are significantly different from one another (*p* < 0.05). Quantification of the percent of the area with fibrosis was conducted using Image J software (National Institutes of Health, Bethesda, MD, USA). Scale bar=50 μm.

The microscopic photograph of Masson's trichrome-stained hepatic sections exhibited normal hepatic cells and central vein with no collagen deposition in the control and BISA groups (Figures 3A,B). While, hepatic sections from ducks receiving CdCl_2_ at 37.5 mg/kg diet showed blue-stained collagen deposition in portal areas (Figure 3C). Collagen deposition increased in Cd 75- and Cd 150-groups (Figures 3D,E). In contrast, a marked decrease (*p* < 0.05) in blue-stained collagen deposition was seen in groups receiving CdCl_2_ + BISA. compared with the groups receiving corresponding CdCl_2_ dose alone (Figures 3F–H). The percent of area of collagen deposition in hepatic tissues of all experimental groups is shown in Figure 3I.

### Oxidative stress and antioxidant markers in hepatic tissues

As shown in Table 4, CdCl_2_ administration produced a status of oxidative stress in hepatic tissues as depicted by marked elevation of MDA levels (*p* < 0.05) and reduction in the activities of SOD, CAT and GSH levels in Cd 37.5, Cd 75, and Cd 150 groups, compared to the controls (*p* < 0.05). In contrast, BISA + Cd 37.5, BISA + Cd 75, and BISA + Cd 150 groups had a significant reduction (*p* < 0.05) in the hepatic MDA level when compared to the group receiving the corresponding CdCl_2_ dose alone. Additionally, in ducks receiving both BISA and Cd, the levels of GSH, SOD, and CAT activities were significantly increased (*p* < 0.05), compared with groups-treated with the corresponding doses of CdCl_2_ only.

**Table 4:** Effect of α-bisabolol on hepatic tissue oxidative stress markers in ducks receiving CdCl_2_ in feed for 4 weeks.

**Experimental groups**	**Parameters**			
	**MDA level**	**SOD activity**	**CAT activity**	**GSH content**
	**(nmol/g tissue)**	**(U/g tissue)**	**(U/g tissue)**	**(mg/g tissue)**
C	28.40 ± 2.02^d^	45.30 ± 3.34^ab^	2.70 ± 0.17^a^	24.48 ± 1.80^ab^
BISA	26.75 ± 1.47^d^	51.50 ± 2.79^a^	2.85 ± 0.18^a^	26.18 ± 1.59^a^
Cd 37.5	45.97 ± 2.88^bc^	31.47 ± 1.50^cd^	1.6 ± 0.15^c^	16.40 ± 1.46^cd^
Cd 75	57.20 ± 4.98^b^	25.15 ± 1.45^d^	1.27 ± 0.09^c^	12.05 ± 1.14^d^
Cd 150	76.30 ± 3.68^a^	14.53 ± 1.39^e^	0.56 ± 0.07^d^	7.35 ± 0.93^e^
BISA + Cd 37.5	31.00 ± 2.29^d^	42.75 ± 2.15^ab^	2.40 ± 0.13^a^	21.68 ± 1.65^ab^
BISA+ Cd 75	34.85 ± 2.55^cd^	39.80 ± 1.62^bc^	2.28 ± 0.11^ab^	19.28 ± 1.04^bc^
BISA + Cd 150	38.10 ± 2.75^cd^	29.12 ± 3.12^d^	1.70 ± 0.22^bc^	13.86 ± 1.08^cd^

Data are expressed as the mean ± SEM (n = 6).

a−e Different superscripts within each column (in each parameter) represent significant differences (p < 0.05). C, control, BISA; α-bisabolol; Cd 37.5, Cd 75, Cd150; CdCl_2_ was administered at 37.5, 75, and 150 mg/kg feed, respectively, for 4 weeks), MDA, malondialdehyde; SOD, superoxide dismutase; CAT, catalase; GSH, reduced glutathione.

### The mRNA expression of TNF-α, Bax, and Bcl-2 genes

**Figure 4:**
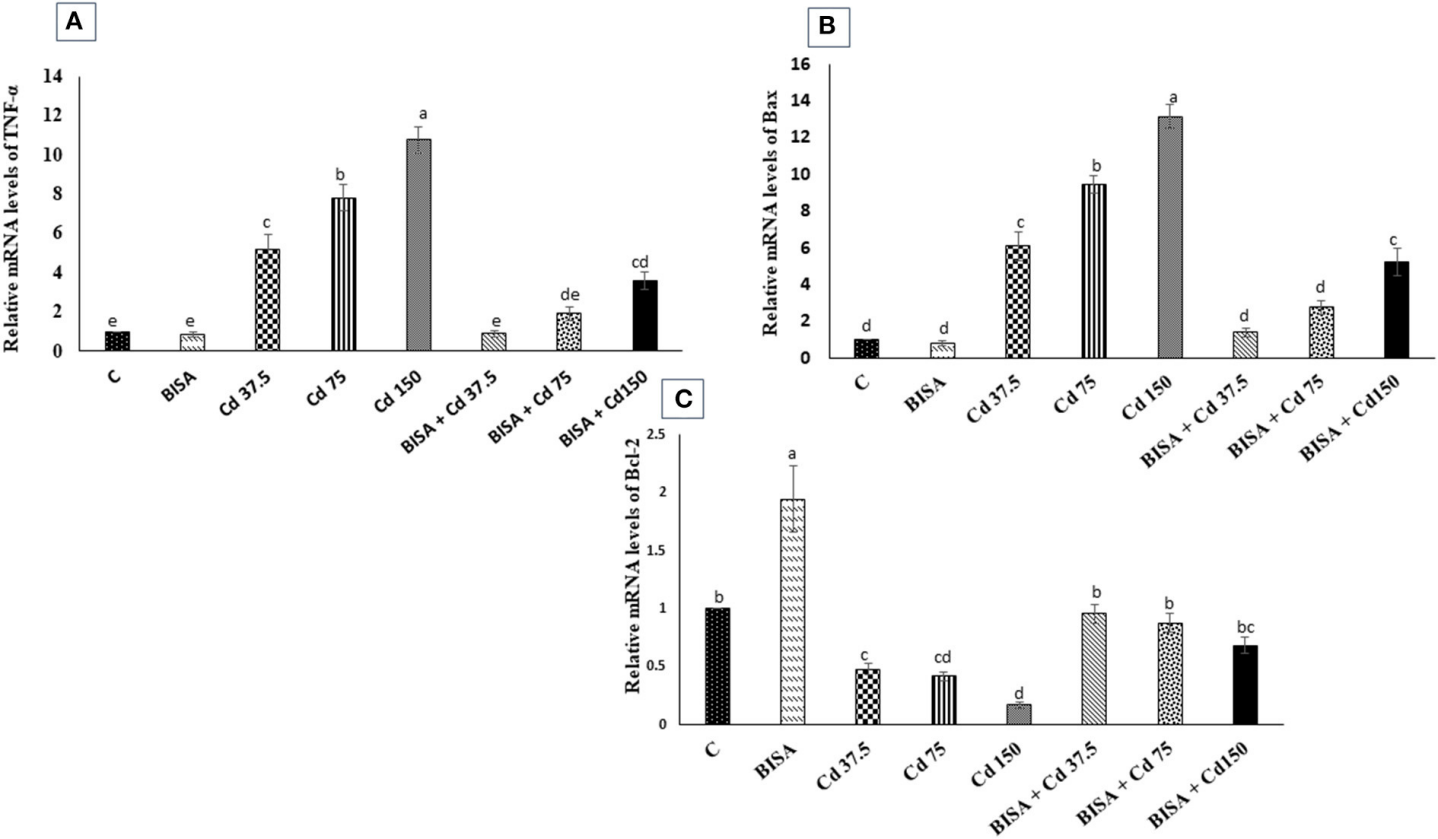
Relative mRNA expression of tumor necrosis factor-α (TNF-α) **(A)**, Bax **(B)**, and Bcl-2 **(C)** in the hepatic tissue of ducks in response to oral administration of α-bisabolol (BISA, 50 mg/kg/day) and/or CdCl_2_ at 37.5, 75, or 150 mg/kg diet) for 4 weeks. Data are displayed as mean ± SEM (*n* = 6 ducks). Bars carrying different letters are significantly different from one another (*p* < 0.05). C, control.

The mRNA expression of a proinflammatory cytokine TNF-α and an apoptotic gene Bax were significantly upregulated (*p* < 0.05) in the hepatic tissue of CdCl_2_-treated ducks, compared to the controls. Conversely, a marked downregulation (*p* < 0.05) in the expression of the hepatic anti-apoptotic gene (Bcl-2) was observed in these ducks, compared to the controls. Also, the mRNA levels of TNF-α and Bax dramatically declined (*p* < 0.05) in ducks receiving BISA + CdCl_2_, compared to those exposed to the corresponding dose of CdCl_2_ only. The expression patterns of TNF-α and Bax genes were completely reversed in BISA + Cd 37.5 and BISA + Cd 75 groups, while they remained significantly higher than the controls in BISA + Cd 150 group. Besides, co-administration of BISA with CdCl_2_ restored the expression levels of Bcl-2 to the level of the controls (Figure 4).

### Immunohistochemical findings of COX-2

**Figure 5:**
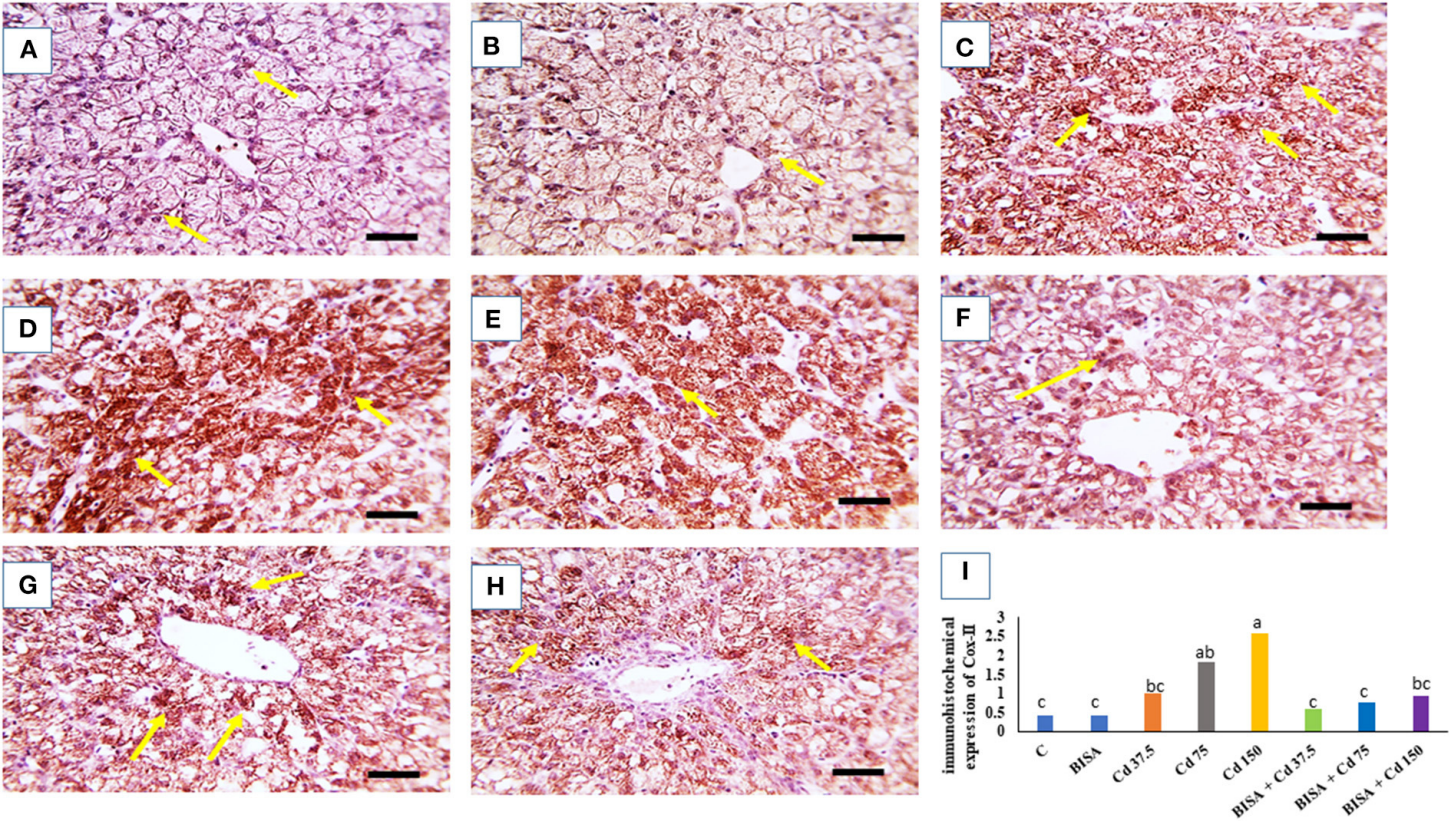
Quantitative data of COX-2 immunohistochemical staining intensity in the duck liver. **(A)** Expresses the control group (C) and **(B)** represents the bisabolol (BISA) alone group; both panels show very mild COX-2 brown staining in hepatocytes. **(C–E)** Are for groups receiving CdCl_2_ at 37.5, 75, and 150 mg/kg diet, respectively, showing a dose-dependent increase in the brown staining. **(F–H)** Represent BISA + Cd 37.5, BISA + Cd 75, and BISA+ Cd 15 groups, respectively, and display a marked decline of COX-2-staining, compared to the corresponding CdCl_2_ alone groups. **(I)** Shows the quantitative data of COX-2 staining in hepatic tissues for the experimental groups. Quantitative data are expressed as mean ± SEM (*n* = 6). Bars carrying different letters are significantly different from one another (*p* < 0.05). The Image J software (National Institutes of Health, Bethesda, MD, USA) was used to quantify the intensities of immunostaining. Scale bar = 50 μm.

The microscopic photographs of immunostained hepatic sections for COX-2 demonstrated very mild positive brown staining of COX-2 in the cytoplasm of hepatocytes in the control and BISA groups (Figures 5A,B). The positive brown staining increased with increasing dose of CdCl_2_ (Figures 5C–E). In contrast, ducks receiving BISA + CdCl_2_ displayed remarkable reduction of COX-2 staining, compared to the corresponding CdCl_2_-alone groups (Figures 5F–H). Figure 5I elucidated the quantitative data of COX-2 staining in hepatic tissues for the experimental groups.

## Discussion

The current research investigated the toxicological effects of CdCl_2_ at 3 doses (37.5, 75, and 150 mg/kg feed), on the hepatic tissues and the role of BISA in antagonizing Cd intoxication in ducks. The present study revealed overt adverse clinical signs in ducks after CdCl_2_ administration at 75 and 150 mg/kg. These observations are in accordance with those of Singh et al. (40) who reported similar clinical signs of toxicity in broiler chickens receiving CdCl_2_ at 50 mg/L in drinking water. These clinical signs may be due to the immunosuppressant effect of Cd which subsequently increased the bird's susceptibility to stress and disease (40). Furthermore, our results showed a marked reduction in BWG, and an increase in the mortality rate in CdCl_2_-treated ducks. Similarly, Erdogan et al. (41) reported a decline in BWG in broiler chickens receiving CdSO_4_ at 25 mg/L drinking water for 6 weeks. Also, Bharavi et al. (28) revealed a drastic drop in BWG in chickens receiving CdCl_2_ at 100 mg/kg feed for 28 days. Moreover, our results showed that the exposure of ducks to CdCl_2_ at 37.5 and 150 mg/kg feed for 4 weeks caused marked hepatic injury as witnessed by elevated serum ALT activity and bilirubin level. These findings were consistent with those of Renugadevi and Prabu (42) who reported an elevation in the serum ALT activity and total bilirubin level in rats receiving CdCl_2_ orally at 5 mg/kg bw/day for 4 weeks.

In the present study, the histopathological investigation of hepatic sections collected from ducks receiving CdCl_2_ at these doses manifested the Cd-induced hepatic damage by showing hydropic degeneration in hepatic cells, occluded sinusoids, congested central veins, and perivascular fibrosis. These structural changes were consistent with those of Li et al. (43) who reported inflammatory cell infiltration, hepatocyte degeneration, and necrosis in the liver of chickens receiving CdCl_2_ in the diet at 140 mg/kg for 60 days. Similarly, ballooning degeneration, coagulative necrosis, and cytoplasmic vacuolation were detected in the liver of chickens treated with CdCl_2_ at 150 mg/kg diet for 60 days (27). Moreover, Koyu et al. (44) reported that CdCl_2_, even in small amount (15 mg/L of drinking water for 30 days) in rats, caused marked pathological alterations in hepatic tissues. In the present duck study, co-administration of BISA with CdCl_2_ enhanced BWG, increased survival rate, restored the normal levels of serum hepatic indices and alleviated the histopathological damage induced by CdCl_2_. Our results were consistent with those of Amaral et al. (45) who reported that BISA ameliorated the hepatic injury induced by acetaminophen in mice. Other sesquiterpenoids, e.g., β-caryophyllene and Rutaecarpine, have a protective effect against the hepatic damage caused by carbon tetrachloride and thioacetamide, respectively (46, 47).

Oxidative stress is the main mechanism underlying the hepatic injury caused by Cd (9). The present study ascertained the capability of Cd to disturb the oxidant-antioxidant balance, evidenced by the increased MDA level, and reduced the anti-oxidant activities of SOD and CAT and the content of GSH in hepatic tissues of ducks. MDA is the main metabolite of lipid peroxidation, which indicates the extent of oxidative damage (48). While, SOD and CAT represent the first line defense antioxidants which play a central role in scavenging free radicals (49). In addition, GSH, a sulfhydryl peptide, is widely distributed in all cellular systems. It participates in the battle against the oxidative insult either by acting as a non-enzymatic antioxidant through direct interaction of its sulfhydryl group with ROS or by acting as a cofactor in the enzymatic detoxification reactions (42). Zhang et al. (50) revealed that Cd has the ability to interact with sulfhydryl (-SH) groups of ROS quenching molecules such as glutathione-SH which ultimately causes indirect elevation of ROS and oxidant-antioxidant imbalance. Our findings were in concordance with those of prior studies (43, 51, 52). In contrast, administration of BISA to CdCl_2_-poisoned ducks showed marked reduction in MDA level and elevation in the activities of SOD, GSH and CAT. Our findings suggested that BISA decrease the liberation of free radicals and restrain oxidative stress. Corroborating with our findings, several authors have reported the antioxidant activity of BISA (15, 19). Additionally, Vinholes et al. (53) attributed the hepatoprotective effect of sesquiterpenoids, including BISA to their lipophilic and polar characteristics which enhance membrane stability and suppress lipid peroxidation. Furthermore, terpenoids enhancing the activity of SOD, consequently increasing the elimination of free radicals (54).

Cd not only upsets the antioxidant system but also induces inflammatory response (55, 56). Inflammation is regarded as a substantial sequel for oxidative stress (57). As Cd promotes oxidative stress and excessive formation of ROS in the liver, nuclear factor-kappa B (NF-KB) separates from its inhibitory kappa B (IκBα) which enhances the generation of proinflammatory cytokines (56). In this context, Cd-induced hepatic damage is accompanied by the increased production of inflammatory mediators (TNF-α, IL-6, IL-1β) (43). The present work showed a remarkable upregulation of mRNA of TNF-α in hepatic tissues of ducks treated with CdCl_2_. Moreover, the immunohistochemical screening displayed high COX-2 expression in the liver of CdCl_2_-intoxicated ducks. COX-2 is an enzyme which is activated by proinflammatory cytokines and serves as a principal contributor to prostanoid synthesis as inflammation proceeds (58). In agreement with our findings, Sanjeev et al. (59) demonstrated a marked increase in the expression of COX-2 in hepatic tissues of rats injected with CdCl_2_ subcutaneously at 10 mg/kg/day for 30 days. In addition, we found that BISA displayed antiinflammatory effects by lowering the mRNA level of TNF-α and the expression of COX-2 in hepatic tissues of ducks. These findings were parallel to those of Barreto et al. (16) who reported marked antiinflammatory activity of BISA against carrageenan-induced pleurisy through reducing the production of TNF-α and the migration of leukocytes to the peritoneal cavity. Similarly, Kim et al. (60) demonstrated that BISA ameliorated the inflammation caused by lipopolysaccharide in RAW264.7 macrophages *via* decreasing the expression of COX-2 and inducible nitric oxide synthase.

Apoptosis is another adverse consequence of oxidative stress; it is needed for cellular turnover and homeostasis (61). Previous studies have denoted that Cd could promote apoptosis by provoking the mitochondrial pathway (52). Liu et al. (62) reported that Cd caused leakage in the mitochondrial membrane, inducing translocation of cytochrome C and apoptosis-inducing factors into the cytosol, leading to caspase cascade and eventually apoptosis. Cd-induced oxidative insult plays a prominent role in its ability to cause apoptosis (63). In the present study, CdCl_2_ intoxication with 3 different dose regimens resulted in significant upregulation of pro-apoptotic gene Bax and downregulation of anti-apoptotic gene Bcl-2 in the liver of ducks. Bax and Bcl-2 are components of Bcl-2 family, responsible for the regulation of the mitochondria-based apoptotic pathway (64). Our findings were consistent with those of prior studies which demonstrated the occurrence of apoptosis in hepatic tissues of chickens intoxicated with Cd (27, 52). In addition, Habeebu et al. (65) reported a dose-dependent apoptosis in the liver of mice exposed to Cd. Furthermore, Cd has been shown to increase the mRNA level of Bax and reduce the mRNA level of Bcl-2 in chicken testis (55).

In the present study, we found that BISA antagonized Cd-induced apoptosis as evidenced by downregulating Bax and upregulating Bcl-2 genes expression in hepatic tissues of ducks. Similarly, Shanmuganathan et al. (66) demonstrated the anti-apoptotic effect of BISA against amyloid-β-induced neurotoxicity in PC12 cells.

In the present study, we found that oral administration of CdCl_2_ for 4 weeks increased the hepatic content of Cd. CdCl_2_ supplementation to the diet of birds (ducks, broilers, cocks, and hens) causes an increase of Cd content in the liver (67, 68). In our study, concurrent administration of BISA with CdCl_2_ dramatically reduced Cd content of the liver in ducks. This effect is probably due to reduction in the active transport of Cd in the gastrointestinal tract (GIT) mucosa or an increase in the excretion of Cd from the GIT or kidneys. Further studies are warranted to investigate the mechanism by which BISA decreases the hepatic Cd content.

## Conclusion

**Figure 6:**
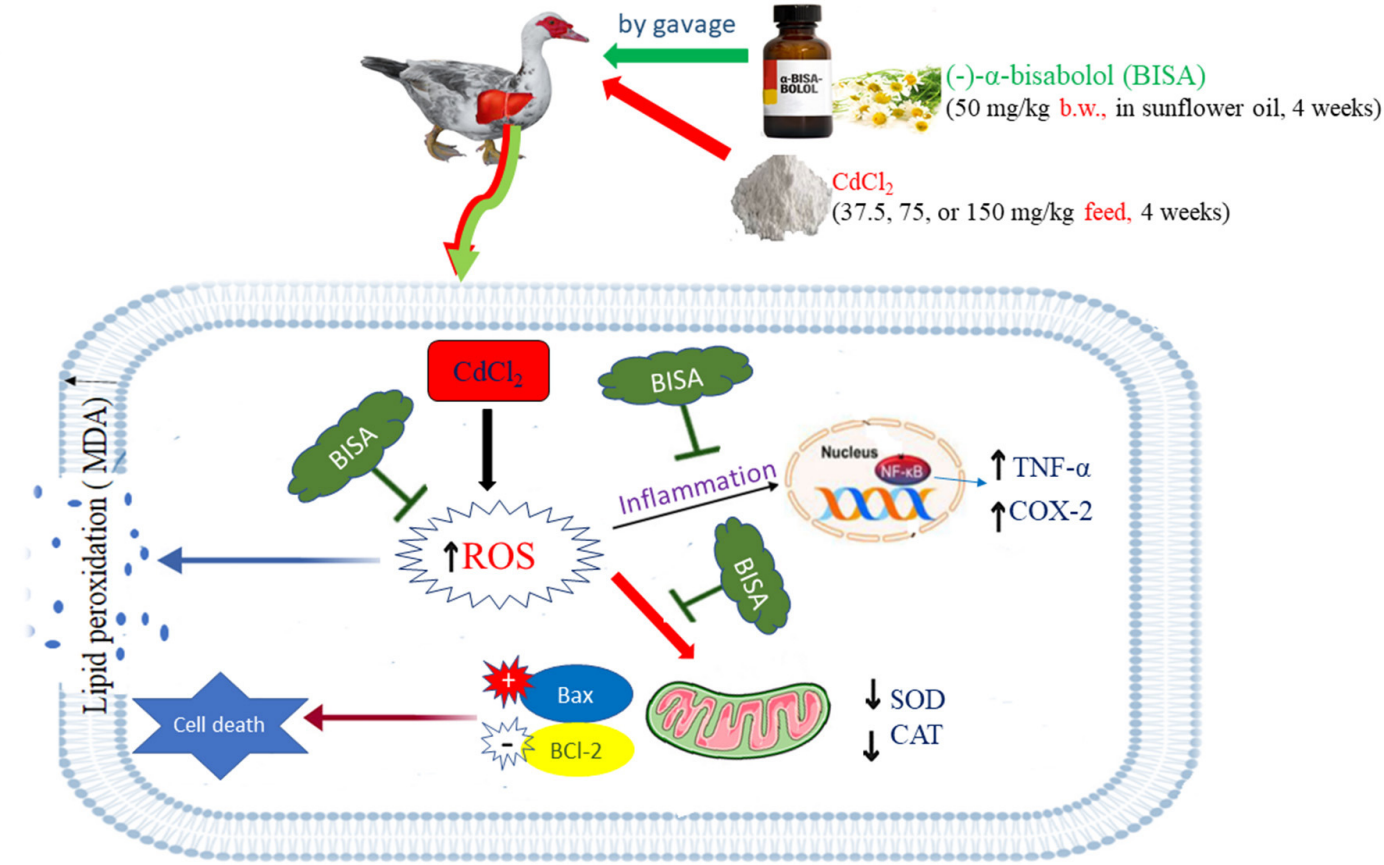
The possible mechanisms of BISA-induced protection against Cd-induced liver toxicity in ducks. The schematic diagram elucidates that BISA exerts its effects *via* antagonizing the oxidative insult, inflammation, and apoptosis caused by CdCl_2_ administration.

This study verified the dose-dependent hepatotoxic effect of CdCl_2_ at 3 doses (37.5, 75, and 150 mg/kg diet) for 4 weeks in ducks. BISA conferred the protection against hepatoxicity induced by CdCl_2_ through reducing the hepatic content of Cd as well as antagonizing oxidative insults, inflammation, and apoptosis. BISA even decreased Cd-induced mortality. Future studies are warranted to gain deeper insight into the molecular mechanisms underlying the antagonistic effect of BISA on Cd-induced hepatic damage. Nonetheless, BISA exerts a great potential to be used as an antidote in the control of Cd poisoning. The proposed mechanisms of BISA in antagonizing CdCl_2_-induced hepatic injury in ducks are presented in Figure 6.

## Data availability statement

The original contributions presented in the study are included in the article/Supplementary materials, further inquiries can be directed to the corresponding author.

## Ethics statement

The animal study was reviewed and approved by Medical Research Ethics Committee of the Faculty of Veterinary Medicine, Mansoura University, Mansoura, Egypt.

## Author contributions

SE: methodology, data curation, and writing the original draft of the manuscript. WH: conceptualization, data curation review, and editing the manuscript. All authors have read and approved the final manuscript.

## Conflict of interest

The authors declare that the research was conducted in the absence of any commercial or financial relationships that could be construed as a potential conflict of interest.

## Publisher's note

All claims expressed in this article are solely those of the authors and do not necessarily represent those of their affiliated organizations, or those of the publisher, the editors and the reviewers. Any product that may be evaluated in this article, or claim that may be made by its manufacturer, is not guaranteed or endorsed by the publisher.

## Abbreviations

ALT: Alanine aminotransferase
BISA: α-bisabolol
BWG: Body weight gain
CAT: Catalase
Cd: Cadmium
COX-2: Cyclooxygenase-2
FBW: Final body weight
GSH: Reduced glutathione
IL-1β: Interleukin-1β
IL-6: Interleukin-6
NF-KB: nuclear factor-kappa B
ROS: Reactive oxygen Species
SOD: Superoxide dismutase
TNF-α: Tumor necrosis factor-α.
