# Supplementary material for: Antagonism of cadmium-induced liver injury in ducks by α-bisabolol
Source: Front Vet Sci. 2022 Nov 7;9:1024549. doi: 10.3389/fvets.2022.1024549 (PMC9676494; doi:10.3389/fvets.2022.1024549)
Supplement: Supplementary file 1 [file Table_1.docx]

Supplementary Material

**Table S1.** Constituents and chemical composition of the basal diet of ducks

| **Ingredients** | **Experimental diets** | |
| --- | --- | --- |
|  | **Starter (0-14d)** | **Grower-finisher (15-42d)** |
| Yellow corn | 49 | 60 |
| Soybean meal, 44% | 33 | 21 |
| Wheat bran | 8.3 | 12.3 |
| Corn gluten, 62% | 4 | 0 |
| Vegetable oil | 2.50 | 3.50 |
| Calcium carbonate | 0.8 | 0.8 |
| Calcium dibasic phosphate | 1.5 | 1.5 |
| DL-Methionine, 98% | 0.1 | 0,1 |
| Vitamin mineral premix* | 0.50 | 0.50 |
| Sodium chloride | 0.3 | 0.3 |
| Total, % | 100 | 100 |
| **Calculated composition** |  |  |
| **DE, Kcal/ kg**** | 2939.62 | 3001.96 |
| **CP, %** | 22.25 | 16.11 |
| **EE, %** | 2.72 | 3.07 |
| **CF, %** | 3.79 | 3.55 |
| **Ca, %** | 1.02 | 1.00 |
| **P, %** | 0.35 | 0.33 |
| **Lysine, %** | 1.18 | 0.84 |
| **Methionine, %** | 0.46 | 0.36 |

DE, digestable energy; CP: Crude protein; EE, ether extract; CF, crude fibre; Ca, calcium; P, Phosphorus

** DE, digestible energy was calculated according to (69)
